# Relationship between maternal age and anogenital distance in patients with primary hypospadias: A case-control study

**DOI:** 10.1080/2090598X.2020.1831425

**Published:** 2020-10-09

**Authors:** Khaled M. Abdelhalim, Ahmed I. El-Sakka

**Affiliations:** Department of Urology, Suez Canal University, Ismailia, Egypt

**Keywords:** Anogenital distance, hypospadias, maternal age

## Abstract

**Objective:**

To evaluate the correlation between maternal age and anogenital distance (AGD) in patients with hypospadias.

**Patients, subjects and methods:**

A total of 82 participants were divided into two groups, Group 1 included 52 male children with different types of primary hypospadias and Group 2 included 30 normal controls. In both groups, child age and weight, maternal age, and AGD were recorded. In Group 1, the Glans-Urethral Meatus-Shaft score was used to categorise the patients into mild (score 3–6), moderate (score 7–9) and severe (score 10–12) hypospadias.

**Results:**

Both groups were similar for maternal age, child age and child weight (*P* = 0.308, *P* = 0.283 and *P* = 0.664, respectively). The mean (SD) AGD was 4.64 (1.23) and 5.33 (1.01) cm for groups 1 and 2, respectively (*P* = 0.011). Patients in Group 1 were subdivided regarding severity of hypospadias into mild (40.4%), moderate (38.5%) and severe (21.1%). There was a significant relationship between older maternal age and short AGD in both groups (*P* < 0.001 for Group 1 and *P* = 0.001 for Group 2). In Group 1, there was a significant correlation between both short AGD and older maternal age, and severe hypospadias (both *P* < 0.001). Maternal age of ≥34 years significantly predicted a severe hypospadias score (10–12) (sensitivity 100% and specificity 68.3%).

**Conclusion:**

Older maternal age is associated with a shorter AGD in patients with hypospadias and controls. Maternal age of ≥34 years is significantly correlated with patients with severe hypospadias.

## Introduction

Hypospadias is a common congenital anomaly with several theories to explain its aetiology [1,2]. Endocrine disruptors, genetic defects and arrest of development are some of the proposed theories for hypospadias. Androgen disturbance has been suggested as a possible aetiology in nearly 30% of patients with more severe hypospadias. *In vitro* fertilisation (IVF) and female’s exposure to progesterone increase the likelihood of having male children with hypospadias [1,2].

The anogenital distance (AGD) is defined as the distance between the mid-anus and the base of the scrotum in lithotomy position. The AGD is dihydrotestosterone dependent and is used as a measure of genital development and also androgen status. Male infants and toddlers have longer AGDs than female infants and toddlers, respectively. Androgen disruptor agents in animal models can lead to abnormal genital lengths. Moreover, exposure to certain phthalates in rodents can suppress fetal androgen levels and disturb both testicular size and Sertoli cell function [3,4].

Endocrine disruptors during pregnancy, particularly in the first trimester, are associated with shorter AGDs in male children suggesting *in utero* male genital maldevelopment. Moreover, boys with complex genital anomalies, such as hypospadias and undescended testicles (UDT), have shorter AGDs compared to boys without genital anomalies [5,6].

Although several studies have shown that older maternal age is a risk factor for hypospadias and also associated with severe types [7]; the association between maternal age and AGD in both hypospadias and normal controls has not yet been well investigated. These findings, in addition to the fact that there is an absence of predicted risk factors of anatomical severity of hypospadias before pregnancy, prompted us to investigate in the present study the association between maternal age and AGD in both children with hypospadias and normal controls.

## Patients, subjects and methods

This case-control study included 52 boys with primary hypospadias who constituted Group 1 vs Group 2 comprised of 30 normal controls. The study was carried out at the Urology Department, Suez Canal University Hospital from January 2018 to July 2019.

The maternal age was recorded in both groups. All the included women had unremarkable pregnancy histories with a single full-term baby and ‘average birth weight’ as stated by the mothers to exclude any selection bias or associated congenital anomalies. The AGD was measured in both groups from the mid-anus to the base of the scrotum in lithotomy position using a ruler.

The Glans-Urethral Meatus-Shaft (GMS) score is based on anatomical features (glans shape/urethral plate width, meatal site, and chordee degree) with a total score of 12 points. The ‘G’ score assesses glans shape and urethral plate width. The ‘S’ score is determined by simultaneously pushing the dorsal and ventral penile skin downward at the peno-pubic and peno-scrotal junction, respectively, to assess the chordee degree, if present. No measuring devices were utilised. The highest ‘M’ score was 4 for proximal penile shaft and peno-scrotal hypospadias. The patients with hypospadias were classified into three categories: mild (GMS score 3–6), moderate (GMS score 7–9), and severe hypospadias (GMS score 10–12) [8–10].

### Exclusion criteria

Patients with past history of penile operations, previous hormonal treatment (local testosterone or human chorionic gonadotrophin injections) or associated other congenital anomalies, e.g. cryptorchidism, were excluded from the study.

### Data analysis

Data were analysed using the Statistical Package for the Social Sciences (SPSS®), version 21.0 (SPSS Inc., Chicago, IL, USA). The descriptive data were presented as percentages and frequencies. The *t*-test was used to compare means of the tested two variables of normally distributed parameters. Pearson correlation was used to test the correlation between maternal age and AGD in both groups. Spearman’s correlation coefficient was used to assess correlation between the GMS score and both AGD and maternal age in Group 1. A *P* < 0.05 was considered statistically significant.

## Results

The mean (SD) age of the boys was 3.12 (1.45) and 2.77 (1.33) years in groups 1 and 2, respectively (*P* = 0.283). The mean (SD) weight of the boys was 13.58 (3.23) and 13.27 (2.87) kg in groups 1 and 2, respectively (*P* = 0.664). The mean (SD) maternal age was 30.58 (7.86) and 28.73 (7.78) years in groups 1 and 2, respectively (*P* = 0.308). Indicating, no statistical difference between the groups for maternal age, and child age and weight (Table 1).

**Table 1.** Descriptive data of both groups

Variables, mean (SD)	Group 1(Hypospadias) *N* = 52	Group 2 (Control) *N* = 30	*P*^a^
Maternal age, years	30.58 (7.86)	28.73 (7.78)	0.308
Child age, years	3.12 (1.45)	2.77 (1.33)	0.283
Child weight, kg	13.58 (3.23)	13.27 (2.87)	0.664
AGD, cm	4.64 (1.23)	5.33 (1.01)	0.011

^a^Student’s *t*-test

The mean (SD) AGD was 4.64 (1.23) and 5.33 (1.01) cm in groups 1 and 2, respectively (*P* = 0.011), indicating that the AGD was significantly shorter in the boys with hypospadias vs the normal controls (Table 1).

**Figure 1.**
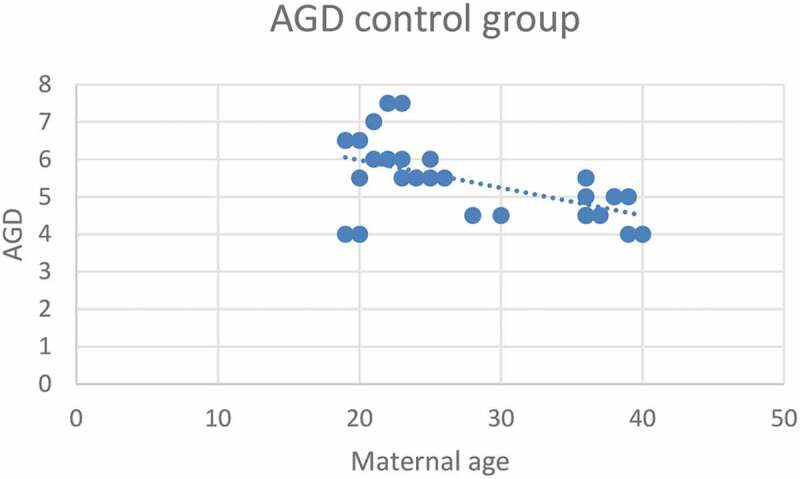
Correlation between maternal age (years) and AGD (cm) in the control group (Group 2)*.*Pearson correlation test, *P* = 0.001

**Figure 2.**
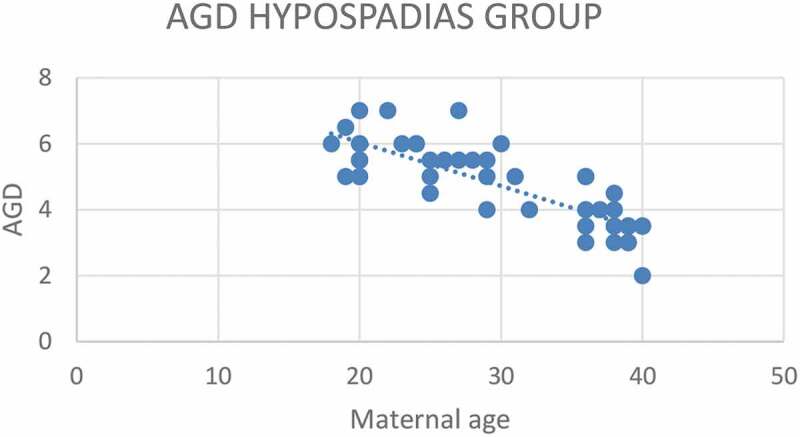
Correlation between maternal age (years) and AGD (cm) in the hypospadias group (Group 1)*.*Pearson correlation test, ***P*** < 0.001

**Figure 3.**
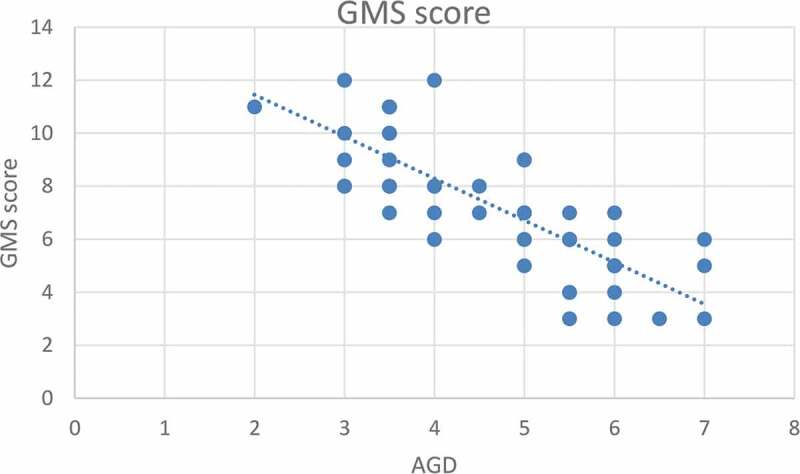
Correlation between AGD (cm) and GMS score for severity of hypospadias*

**Figure 4.**
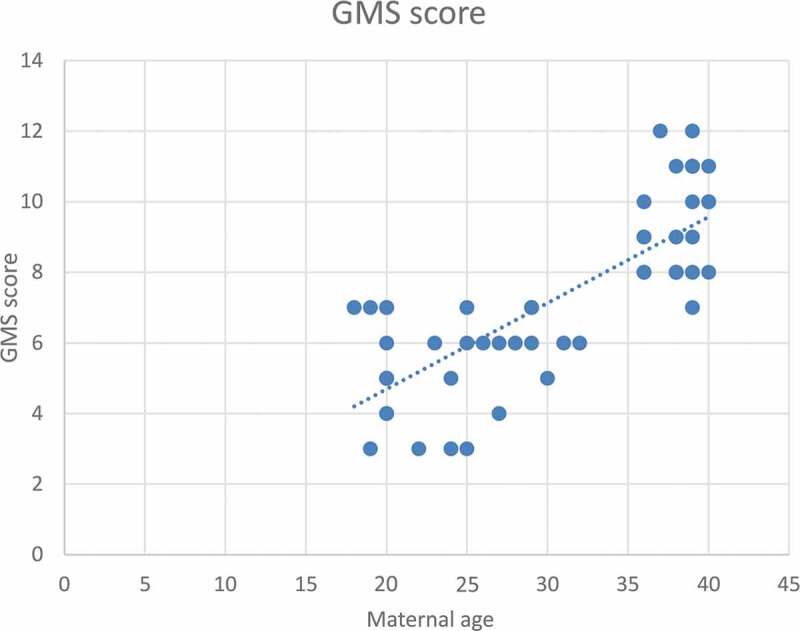
Correlation between maternal age (years) and GMS score for severity of hypospadias*

According to severity of hypospadias and GMS score, patients in Group 1 were subdivided into mild (40.4%), moderate (38.5%) and severe (21.1%), respectively (Table 2). The maternal age was significantly related to the AGD in both groups, with an inverse correlation (*P* < 0.001 for Group 1 and *P* = 0.001 for Group 2; Figures 1 and 2). Short AGD was significantly correlated with a high GMS score in Group 1, with an inverse correlation (*P* < 0.001; Figure 3). In Group 1, older maternal age was strongly associated with a high GMS score, with a positive correlation (*P* < 0.001; Figure 4).

**Table 2.** GMS scores in Group 1 (Hypospadias)

GMS Score, *n* (%)	Group 1 (Hypospadias) *N* = 52
3–6, Mild	21 (40.4)
7–9, Moderate	20 (38.5)
10–12, Severe	11 (21.1)
Total	52 (100)

*Spearman correlation test, *P* < 0.001.

*Spearman correlation test, *P* < 0.001.

In Table 3, regression analysis revealed that maternal age and child age were significantly associated with AGD (*P* < 0.001). Every 1-year increase in the maternal age was associated with 0.07 cm decrease in the AGD. While, every 1-year increase in the child age was associated with 0.26 cm increase in the AGD (multiple linear regression analysis).

**Table 3.** Predicting AGD among all groups^a.^

	β ^b^	Standard Error	*t* ^c^	*P*
(Constant)	6.514	0.507	12.842	0.000
Maternal age (years)	–0.066	0.016	–4.160	0.000
Group (cases vs controls)	1.045	0.597	1.749	0.084
Child age (years)	0.256	0.053	4.794	0.000
Group*maternal age	–0.056	0.020	–2.837	0.006

^a^*R*^2^ = 0.714; Model ANOVA *P* < 0.001

^b^β regression coefficient

^c^Student’s t-test

However, the interaction term between the study group and maternal age was statistically significant, meaning that the association between maternal age and AGD was moderated by being a hypospadias case or control. Therefore, the regression model was repeated on each study group separately. Among hypospadias cases, maternal age was the only significant predictor; every 1-year increase in the maternal age was associated with 0.127 cm decrease in the AGD. In the control group both maternal age and child age were significantly associated with AGD (multiple linear regression analysis) (Table 4).

**Table 4.** Predicting AGD in each group

Models	Predictors	β ^c^	Standard Error	*t* ^d^	*P*
Hypospadias cases ^a^	(Constant)	8.156	0.473	17.230	0.000
	Maternal age (years)	–0.127	0.012	–10.413	0.000
	Child age (years)	0.119	0.066	1.806	0.077
Controls ^b^	(Constant)	5.476	0.405	13.537	0.000
	Maternal age (years)	–0.057	0.011	–5.096	0.000
	Child age (years)	0.538	0.065	8.262	0.000

^a^Model 1: *R*^2^ = 0.72; model ANOVA *P* < 0.001

^b^Model 2: *R*^2^ = 0.81; model ANOVA *P* < <0.001

^c^β regression coefficient

^d^Student’s *t*-test

**Figure 5.**
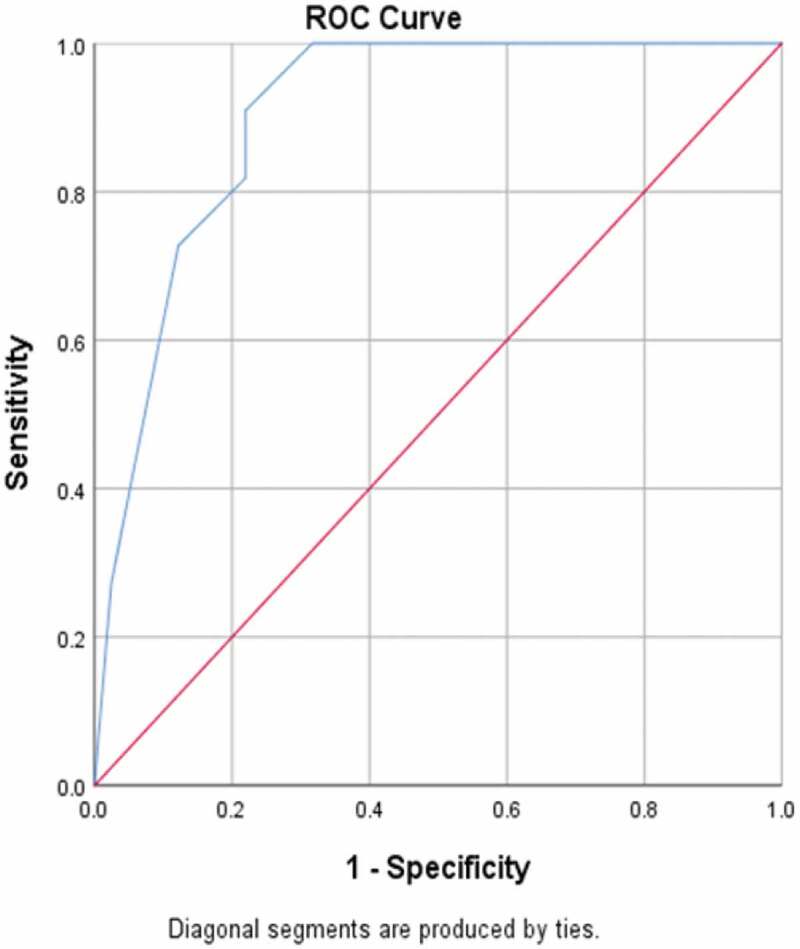
Cut-off value of maternal age for predicting severe GMS score

In Figure 5, receiving operating characteristic (ROC) curve analysis revealed that maternal age significantly predicted severe hypospadias (GMS score 10–12), with a significantly large area under the curve (90.4%) and a highly sensitive cut-off value of maternal age of ≥34 years (sensitivity 100% and specificity 68.3%).

## Discussion

Hypospadias is defined as ventral location of the urethral meatus, which is usually associated with ventral penile curvature and deficient ventral foreskin. Hypospadias is associated with a wide range of severity, from the mild type with glanular urethral opening with deficient ventral preputial development, to the severe type with a perineal opening, an enlarged prostatic utricle and peno-scrotal transposition [11].

Hypospadias is considered a common paediatric congenital anomaly with an incidence of one in 300 live births [2]. Maternal age has been found to be an important risk factor for hypospadias in the past few years. Older maternal age is not only associated with increasing incidence of hypospadias, but is also linked to the most severe hypospadias type [7,12].

Fetal testis maldevelopment at an early gestational age can lead to testicular dysgenesis syndrome (TDS), which includes hypospadias, UDT, testicular germ cell tumour, and oligospermia. The ‘male programming window’ (MPW) is likely to be at 8–14 weeks of gestation during which time genital development is programmed and has been identified a critical period in both animals and humans [13].

Disruption of androgen action only during this MPW period leads to hypospadias and UDT, as well as altered AGD in rat models. The TDS disorders based on this fact may originate from androgen disruption during the MPW, and AGD may be considered a predictor for early fetal androgen action [13].

Hypospadias prevention should include genetic counselling before pregnancy and minimising maternal exposure to harmful endocrine disruptor agents that affect male urethral development, especially during the first trimester of pregnancy [2,11].

The relationship between AGD and serum testosterone in both genders has been reported, with a long AGD associated with elevated serum testosterone levels [14]. In addition, it has been thought that a shorter AGD is associated with infertility later in adult life. Furthermore, a shorter AGD has been reported to be associated with severe anatomical forms of hypospadias. This may explain the unfavourable outcome of complex repair in children with severe types of hypospadias [14,15].

In general, male infants and toddlers have longer AGDs than female infants and toddlers, respectively. Androgen disruptor agents can lead to abnormal genital lengths in animal models [3,4]. Women exposed to endocrine disruptors during early pregnancy have male children with shorter AGDs, suggesting an early fetal male genital maldevelopment [5,6].

The mean (SD) maternal age in our present population was 30.58 (7.86) years in Group 1 and 28.73 (7.78) years in Group 2 (*P* = 0.308), thus no statistically significant difference between the groups with homogenous distribution and good randomisation. The explanation for older maternal age may be related to various factors, e.g. older age at marriage, families may elect to defer childbearing, preference of multiparity even for women of advanced age, primary or secondary female or male factor infertility, and finally, advances in assisted reproductive technology (ART) that helps women to get pregnant despite their advanced age.

The mean (SD) age of the boys was 3.12 (1.45) years in Group 1 and 2.77 (1.33) years in Group 2 (*P* = 0.283) and their respective weights were 13.58 (3.23) and 13.27 (2.87) kg (*P* = 0.664), indicating that age and weight were similar in the two groups minimising their impact on AGD measurements. A previous experimental animal model study showed that in male rats the AGD increased by 0.26 mm for each 1 g increase in body weight. In females, the AGD increased 0.13 mm per 1 g increase in body weight [16]. Therefore, the gestational age and birth weight might be important risk factors for AGD.

The mean (SD) AGD was 4.64 (1.23) cm in Group 1 and 5.33 (1.01) cm in Group 2 (*P* = 0.011), indicating that the AGD was significantly shorter in the boys with hypospadias compared with the normal controls. As both the age and weight were homogenously distributed between the two groups, it is possible that the shorter AGDs in Group 1 may reflect the endocrine action disruption theory in the MPW and be the cause of the hypospadias and UDT. This finding was similar to that reported by Hsieh *et al*. [6], who stated that boys with complex genital anomalies, such as hypospadias and UDT, had a significantly shorter AGDs compared to boys without any genital anomalies [5,6,13].

According to severity of hypospadias and GMS score, patients in Group 1 were subdivided into mild (40.4%), moderate (38.5%) and severe (21.1%) groups, respectively. It was postulated that a high GMS score might reflect the postoperative complication rate, particularly the likelihood of urethrocutaneous fistula. Preoperative GMS score assessment may aid in clinical decision-making, operative technique planning and parental counselling [17,18].

Maternal age was significantly related to a short AGD in both groups, with an inverse correlation (*P* < 0.001 for Group 1 and *P* = 0.001 for Group 2; Pearson correlation coefficient) and a highly significant correlation in Group 1 compared to Group 2, and this may be explained by the shorter AGD in Group 1 vs Group 2. Using regression analysis, in the control group, every 1-year increase in the maternal age was associated with 0.057 cm decrease in the AGD. While in the hypospadias group (Group 1), every 1-year increase in the maternal age was associated with 0.127 cm decrease in the AGD. The AGD is known as an indicator of prenatal androgen exposure and suggests an androgen deficit as a possible theory of hypospadias incidence. A shorter AGD and severe hypospadias is associated with more challenging repair and a higher complication rate of hypospadias surgery, which is important to discuss during maternal counselling. Furthermore, the effect of older maternal age with its correlation to the child’s AGD may indicate possible subfertility. In a previous study, the AGD was significantly correlated with sperm density and total motile sperm count. After adjusting for demographic and reproductive variables, for each 1-cm increase in a man’s AGD, the sperm density increases by 4.3 million sperm/mL (95% CI 0.53–8.09, *P* = 0.03) and the total motile sperm count increases by 6.0 million [3]. In addition, these data have a very important clinical implication for maternal counselling, especially in the era of IVF and delayed marriage age.

The increased incidence of ART and maternal exposure to endocrine disruptors during pregnancy, particularly in the first trimester, could explain this finding due to decreased androgen impact on external genitalia during development, which results in a short AGD.

A short AGD was significantly correlated to a high GMS score in Group 1, with an inverse correlation (*P* < 0.001). This correlation may be due to diminished prenatal androgen exposure, which causes both a short AGD and high GMS score. In addition, in Group 1 older maternal age was significantly correlated with a high GMS score, with a positive correlation (*P* < 0.001). In addition, ROC curve analysis revealed that maternal age significantly predicted severe hypospadias (GMS score 10–12), with a highly sensitive cut-off value of a maternal age of ≥34 years (sensitivity 100% and specificity 68.3%).

Similar results were reported by Carlson *et al*. [7] and Fisch *et al*. [12], who stated that older maternal age was correlated with the increased likelihood of hypospadias, particularly severe types, without any specific explanation for this correlation. A short AGD is associated with both severe hypospadias and a substantial diminution of the healing potential and thus less favourable repair outcomes [7,12].

Mothers aged ≥34 years should be counselled about the increased likelihood of hypospadias in their children, especially severe types, which necessitates more complex techniques for repair and a high rate of postoperative complications. Future study is needed to compare mothers aged >34 years with a history of IVF or progesterone intake and those with unremarkable pregnancy histories to determine the effect of these risk factors on the AGD.

A short AGD was associated with both older maternal age and high GMS scores, and thus more risk of postoperative complications. Extrapolation of the present study results may open the door to finding a way to reduce or even prevent hypospadias, especially in older mothers, by enhanced fetal perineal growth and avoidance of any endocrine disruptor agents, especially in the first 16 weeks of gestation. Moreover, the increasing age of marriage and utilising of IVF techniques has made this problem more prevalent, which necessitates partner counselling before pregnancy.

Limitations of the present study include a relatively small sample size and the lack of hormonal assessment in the mothers and children, which might help to explain the correlation between maternal age and hypospadias. The present study provides a basis for further studies to investigate the effect of androgen exposure and the importance of avoiding any endocrine disruptors, especially in more elderly mothers, who have a high risk of having children with severe hypospadias.

## Conclusion

Older maternal age is associated with a shorter AGD in patients with hypospadias and controls. Maternal age of ≥34 years is significantly correlated with patients with severe hypospadias.
